# Resveratrol and Gut Microbiota Synergy: Preventive and Therapeutic Effects

**DOI:** 10.3390/ijms242417573

**Published:** 2023-12-17

**Authors:** Milos Gostimirovic, Jovana Rajkovic, Ana Bukarica, Jovana Simanovic, Ljiljana Gojkovic-Bukarica

**Affiliations:** 1 Department of Cardiovascular Pharmacology, Institute of Pharmacology, Clinical Pharmacology and Toxicology, Faculty of Medicine, University of Belgrade, 11129 Belgrade, Serbia; milos.gostimirovic@med.bg.ac.rs (M.G.); jovana.simanovic@rfzo.rs (J.S.); bukarica@rcub.bg.ac.rs (L.G.-B.); 2 Institute for Cardiovascular Diseases Dedinje, Faculty of Medicine, University of Belgrade, 11040 Belgrade, Serbia; abukarica@icloud.com

**Keywords:** resveratrol, Mediterranean diet, gut microbiota, cardiovascular diseases, diabetes mellitus, obesity, quality of life, physical activity, post-COVID-19 syndrome

## Abstract

The role of an imbalanced high-fat diet in the pathophysiology of common chronic noncommunicable diseases has been known for years. More recently, the concept of ‘gut microbiota’ and the interaction between their composition and gut metabolites produced from the intake of dietary products have gained the focus of researchers, mostly from the perspective of the prevention of cardiovascular and metabolic disorders, which are still the leading cause of death globally. The aim of this work is to highlight the health benefits of the interaction between resveratrol (RSV), red grape polyphenol, and gut microbiota, through aspects of their therapeutic and preventive potentials. Since changed microbiota (mostly as a consequence of antibiotic overuse) contribute to the persistence of post (‘long’)-COVID-19 symptoms, these aspects will be covered too. Data were obtained from the electronic databases (MedLine/PubMed), according to specific keywords regarding the protective role of resveratrol, the gut microbiota, and their synergy. RSV exerts beneficial properties in the modulation of cardiovascular, metabolic, and post-COVID-19-related disorders. In healthy individuals, it maintains an ergogenic capacity, prevents oxidative stress, and modulates the inflammatory response. Overall, it improves quality of life. The RSV–gut-microbiota interaction is beneficial in terms of maintaining human health. Along with physical activity, it is key for the prevention of chronic noncommunicable diseases.

## 1. Introduction

The continuous development of molecular science and the increased influence of external risk factors have added new insights to the knowledge about the etiology of many diseases, while the improvement in health services has reduced the burden of their complications and led to a longer life expectancy. However, hectic and harmful lifestyles, unstable socioeconomic factors, extreme climate change and, until recently, COVID-19-related psychosomatic disorders are having negative impacts on human health, especially in the interplay with the common comorbidities such as cardiovascular diseases (CVD) [1] and diabetes mellitus (DM) [2]. According to the most recent data, in 2019, more than 550 million people suffered from CVD, with a mortality rate of nearly 18 million people (31% of global deaths). Over the past two decades, an increased incidence has been noticed not only in industrialized counties but also in developing countries, especially the Eastern Mediterranean region [3]. In the Republic of Serbia (RS), CVD is ranked first in terms of mortality, with a share of 47.3% of total deaths (or 51,624 people) in 2022. Among CVD, 18.3% (or 9448 people) died from ischemic heart diseases, out of which 48.3% (or 4565 people) died of acute coronary syndrome [4]. Similarly, the global prevalence of DM in 2017 was 462 million people (6.3% of the world’s population), with a prevalence rate of 6059/100,000 people and an expected projection of 7079/100,000 people [5] by 2030. In RS, 3091 people died as a consequence of DM in 2022, with a 2.8% share of the total mortality [4]. These data strongly suggest the need for urgent therapeutic interventions, especially in the post-pandemic era, when the impact of those diseases on global mortality is expected to be stronger than ever. Due to proven health benefits, dietary polyphenols are considered a new therapeutic strategy, especially for common noncommunicable diseases (CNDs). Recently, studies have confirmed their more enhanced synergistic effects after their interaction with the human gut microbiome. One of the risk factors for those diseases is an unbalanced diet, which directly leads to the development of obesity, dyslipidemia, and metabolic syndrome and, indirectly, through changes in the composition of the gut microflora. 

Therefore, the aim of this review is to present the effects of the most studied dietary polyphenol today, resveratrol (RSV), in ameliorating common CNDs such as cardiovascular diseases (CVD) and diabetes mellitus (DM). Special consideration will be given to RSV–gut-microbiota interactions in lowering post-COVID-19 somatic symptoms, which may be debilitating for convalescents. In the end, a note will be given to the impact on healthy people in maintaining proper health and life quality. 

## 2. Resveratrol, Mediterranean Diet, and the Human Gut Microbiome

The knowledge from basic research that scientists have gained regarding the role of RSV in preventing and treating various diseases has been applied in different fields of medicine; hence today, the literature offers many clinical trials in which RSV has proved its efficacy and/or has opened new horizons of research. Although its concentrations may vary among dozens of natural sources, grape berry skin represents the most important one. Having rich and available sources of RSV on one hand and health-improving effects on the other hand, one may speculate that this substance has a potential for far-reaching health benefits, which is why it is still extensively studied. However, a stumbling block in its usage is its inconvenient pharmacokinetics [6], mostly due to the extensive phase II metabolism in the liver, which causes low bioavailability after an oral intake and limited permeation through the blood–brain barrier (BBB) compared to other polyphenols (e.g., epigallocatechin) [7]. 

In order to reduce the impact of an unfavorable pharmacokinetic profile on the expression of its full therapeutic potential and wider clinical use, several strategies are being applied [8]. For example, simultaneous administration with modulators of glucuronidation, methylation of RSV analogs, or RSV’s binding with its endogenous ligands (albumin, LDL lipoprotein, fibrinogen and hemoglobin) have been suggested. In addition, new methods are being investigated to improve the tissue delivery of RSV—a combination with dimethyl beta cyclodextrins (DM-β-CDs), the use of microparticles and vesicles with the potential to bind hydrophilic and hydrophobic substances, and recently, nanosponge formulations (a type of nanoparticle delivery system) [9,10]. Together with food-compatible ways of RSV delivery (for example, peanut oil), many sophisticated methods are represented, and recently, attention has been paid to its analogs with more favorable kinetics, too. Recently, the transport of polyphenols in exosome-containing extracellular vesicles (EEV) was also investigated. Namely, in healthy volunteers, upon oral consumption, glucuronide conjugates of RSV and dihydroresveratrol (but no free RSV) were found in plasma EEV, reaching to local or distant organs, including the BBB, suggesting the role of EEV in RSV’s (central) effects [11]. However, in spite of all the difficulties in obtaining a suitable means of delivery to target tissues, RSV’s inevitable role in modern medicine is not to be questioned.

The Mediterranean diet (MD) represents the consumption of specific food consisting of proper, balanced, and polyphenol-rich substances and is highly recommended by experts for everyday use. The core food of this diet, whole grains, fruits, vegetables, beans, herbs and spices (coriander, rosemary, lavender, saffron, mint, etc.), nuts, red wine, and olive oil, not only maintains overall health and improves quality of life but also ameliorates the burden of many diseases. This is especially important in the time of the massive depopulation seen in the post-COVID-19 pandemic, where the integrity of an overwhelmed country’s health system may be protected or even enhanced by massive preventive policies such as the promotion of a healthy diet. Therefore, the healthy habit of enriching the diet with the concepts of MD is encouraged in every population group, especially in light of the benefits of the interaction between the metabolized constituents of MD and human gut microbiota. 

The human gut microbiome represents a complex ecosystem of trillions of microorganisms including bacteria, fungi, and different types of viruses, mostly bacteriophages. Its composition remains comparatively permanent throughout one’s life and is unique to each individual but shows certain temporary fluctuations depending on the food that is consumed [12]. 

Upon oral intake, RSV is subjected to extensive and dynamic metabolism in the upper gastrointestinal tract (GIT; stomach and small intestine), whereby the activity of different enzymes is converted to more water-soluble absorbable forms. However, it is known today that even unabsorbed portions of RSV play an important role in its overall biological activity, since the majority of RSV-derived metabolites reach the lower GIT, where they interact with gut microbiota. Since the human microbiome is considered the main homeostatic regulator of many processes, this interaction could be useful, especially in ameliorating metabolic syndrome and cardiovascular diseases but also in diseases associated with microbial dysbiosis, such as inflammatory bowel disease, tumors, and obesity [13]. For several gut-microbiota-derived metabolites of RSV—dihydroresveratrol (DHR), lunularin [14] and 3,4′-dihydroxy-trans-stilbene, specific chemopreventive properties in the kidney and colon have been identified [15]. Further, these interactions can be useful for a deeper explanation of its role in the immune response, regulation of bone metabolism and blood rheology, and interestingly, its potential use in a gene therapy. This specific therapeutic use came after a study by Ozog et al. [16], the results of which showed that RSV’s synthetic cyclic metabolite, specifically caraphenol A, was able to enhance lentiviral vector-mediated gene delivery in hematopoietic stem cells. Moreover, it is known that chemotherapies induce negative alteration in the gut microbiota, especially in patients with hematological malignancies, making them subjects for hematopoietic cell transplantation (HCT). This treatment modality, on the other hand, may cause graft-versus-host disease (GvHD), which can further negatively impact the gut. Lacking gut microflora, these patients may be at risk for opportunistic infections, organ failure after HCT, and treatment-related morbidity. Therefore, it is speculated that quantitative and qualitative control of the gut microflora contributes to RSV’s antitumorigenic activity. Lately, a new concept regarding the regulation of gut microflora has attracted attention. The so-called “gut–brain axis” understanding of the disease pathogenesis implies the connection between gastrointestinal function and neuroendocrine disorders in which glucagon-like peptide-1 (GLP-1) plays a central beneficial role. This peptide markedly improves neuronal function, glucose homeostasis, and insulin action both in the peripheral and the central nervous system (CNS). Since those actions require activation of the SIRT-1 (silent mating type information regulator homolog 1) and Akt-dependent FoxO1 pathway, the assumption that RSV could promote the effects of GLP-1 in the intestine and in the brain has led to the development of new studies. Although it was proven in a model of high-fat-fed mice, it is still unclear whether RSV directly affects the release of GLP-1 in humans [17]. However, it is known today that RSV may affect serotonin (5-HT), another neuroprotective factor produced by enterochromaffin cells in the gut, by modulating its release through GLP-1 regulation [17]. Also, bacterial metabolites are known neuromodulators, uremic toxins, and proinflammatory/anti-inflammatory cytokines [12]. Given that intestinal microbes play a role in the development of neurological disorders such as anxiety, autism, depression, and encephalopathy [18], by interfering with diverse cellular signaling including AMPK (adenosine monophosphate-activated kinase), cAMP, and NF-kB (nuclear factor kB) and controlling the balance of 5-HT, RSV may be an effective therapeutic strategy for both CNS and intestinal diseases [17]. Moreover, RSV reverses microbial dysbiosis-induced colitis by suppressing the colonic inflammation caused by CD4^+^ Th_1_/Th_17_ cells [19]. The newest systematic review of 17 randomized control trials that investigated the influence of MD on the gut microbiota suggested, however, that MD did not alter the microbiota diversity and metabolism [20]. 

**Figure 1:**
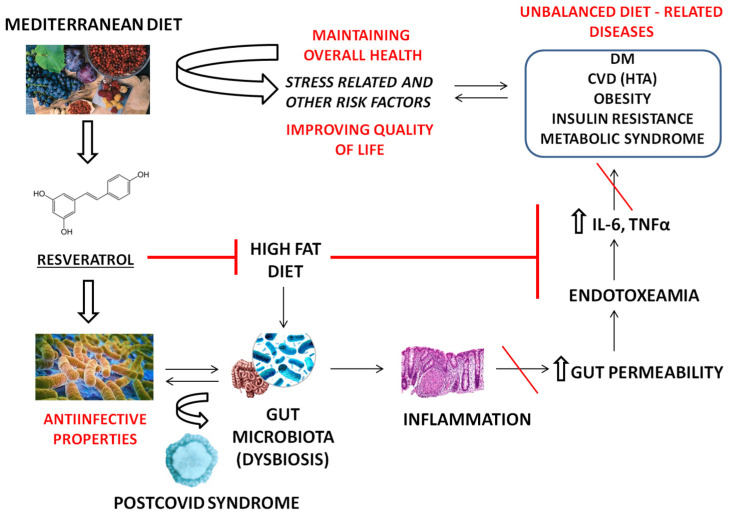
An overview of the properties of RSV on the most common dietary-related diseases. A high-fat diet (HFD) may deregulate the gut microbiome and increase local and systemic inflammation. This contributes to the development of HFD-related diseases, including CVD (cardiovascular diseases). When the gut barriers are impaired, lipopolysaccharides (LPS) of G-negative bacteria bind to Toll-like receptors on the surface of immune cells, additionally triggering inflammation, endotoxemia, and increased gut permeability. As a major component of Mediterranean diet, RSV attenuates the influence of HFD in the pathophysiology of CVD. IL—interleukin, TNF—tumor necrosis factor, HTA—hypertension.

Regarding metabolic diseases, obese subjects who underwent several dietary interventions had the most significant phyla and species changes in microbiota, which revealed the role of *Firmicutes/Bacteroides* spp. in the obesity phenotype. As this concept frequently tends to be misleading due to the lack of association with the host’s lifestyle factors, probably the most useful therapeutic approach represents microbiota changes in combination with a tailored diet and drugs to treat obesity [21]. Figure 1 represents the aspects of RSV’s beneficial effects covered in this review in relation to the health consequences of a high-fat unbalanced diet.

## 3. RSV and Gut Microbiota Synergy in Pathological Conditions

### 3.1. Resveratrol, Gut Microbiota, and Cardiovascular Diseases

Gut microbiota produce biologically active metabolites with distinguished physiological features; thus, any change in its composition may lead to multiple diseases, including CVD [22]. It was shown that a major causative metabolite was TMA—trimethylamine [23], a derivative of dietary nutrients abundantly present in a western diet (lecithin, choline, carnitine, betaine) [24]. Upon digestion, TMA is oxidized through hepatic flavin monooxygenases (FMO) to trimethylamine N-oxide (TMAO), which promotes thrombogenesis, vascular inflammation, kidney fibrosis, and end-organ damage. Notably, the mechanisms of thrombosis include enhanced responsiveness to submaximal stimulation by agonists (thrombin, collagen, ADP) and stimulation of the tissue factor, an activator of the extrinsic clotting pathway [25]. Overall, higher levels of TMAO predict a higher risk for adverse cardiac events. This was proved in a meta-analysis of 25,000 people, where every 10 µmol/L of TMOA was associated with nearly 8% increase in all-cause mortality [26]. Another important metabolite, PAG—phenylacetylglutamine [27], is produced by microbial metabolism of phenylalanine and shows prothrombogenic properties through the stimulation of adrenergic receptors, which are involved in platelet and heart functions [28]. Gut microbiota also enhance bile acid hydrolysis into secondary derivatives, many of which have a hormone-like function, stimulate host nuclear receptors including FXR, LXR, and PXR (farnesyl, liver, and pregnane X) and specific G-protein coupled (TGR5) receptors, which impact the host physiology and enhance the risk for CVD. Through anerobic fermentation of dietary fibers, gut microbiota produce important short-chain fatty acids (SCFA) such as acetate, propionate, and butyrate, which are major energy sources for colonocytes and play a role in neurogenesis, lipid/glucose metabolism, and blood pressure regulation [29]. The antihypertensive effects of SCFAs are mediated through its receptors—Olfr78 (olfactory receptor 78) in the juxtaglomerular apparatus and Gpr41 (G-protein receptor 41) in the vascular endothelium and might be explained by a decline in cardiac output and loss of vascular resistance. Microbiota-derived metabolites such as uremic toxins (p-cresole sulphate, indoxyl sulphate) regulate blood pressure by modulation of the renin–angiotensin–aldosterone system [30]. Based on all the abovementioned, targeting gut microbes in addition to dietary interventions and enhancing the functionality of microbiome by prebiotics represent potentially promising novel therapeutics. 

During the 1990s, the number of papers about RSV’s effects on CVDs started to grow. In the last ten years, more than 100 research papers have been published annually regarding RSV’s impacts on CVDs. During the search for a molecule that can prolong life and improve its quality, RSV has stood out. However, the treatment of CVDs remains a major problem, despite health care improvements, because of a sedentary lifestyle, the low quality of nutrition, and lack of physical activity, which outweigh the benefits that medicine and science have brought. A positive correlation between RSV intake and cardioprotection has been investigated in a large number of animal models as well as in human trials. 

Out of several other herbal supplements (beetroot juice, bergamot extracts, barberry, pycnogenol), RSV represents a phenolic compound with proven blood-pressure (BP) lowering effects, which is recommended to be included in the nutrition of people with hypertension [31]. However, its appropriate dosage is still debatable [32]. A positive association between systolic-BP lowering RSV activity and body-mass index (BMI) at the baseline has been shown especially when used at a high dose (≥300 mg/day) and in diabetic patients [33]. This has been confirmed for both systolic and diastolic pressure in a subsequent novel study [34]. In a study on an experimental model of 2K1C (two-kidney one-clip) rats, renal stenosis and activation of the renin–angiotensin system (RAS) caused renovascular HTA (hypertension) and consequent cardiac hypertrophy; the main aim was to investigate whether RSV alone or in combination with captopril is capable of reducing collagen deposition and ameliorating cardiomyocytes’ remodeling and fibrosis. The results showed that RSV independently of captopril reduced systolic blood pressure and whole heart hypertrophy in 2K1C rats [35]. Preclinical studies related to HTA indicated that a positive effect of RSV [36] on the blood vessels could be achieved through endothelium-dependent and endothelium-independent mechanisms [2,37]. Endothelium-dependent mechanisms include increased production of NO, in which SIRT1, AMPK, and ROS (reactive oxygen species) are proposed as the main mediators [2]. Endothelium-independent mechanisms of RSV include modulation of potassium (K) channels, a reduction in vasoconstrictor molecules (e.g., angiotensin II, endothelin-1), and the inhibition of vascular smooth muscle cell contractility via the inhibition of myosin phosphatase-targeting subunit 1 (MYPT1)/myosin light chain (MLC) phosphorylation [2]. Through in vitro and in vivo animal studies, RSV has confirmed benefits on CVD; so, in recent years, the number of clinical trials has increased. Clinical trials investigate the role of RSV supplementation in the prevention and improvement of CVD including endothelial dysfunction, HTA, peripheral arterial disease, heart failure, etc. Still, expectations of RSV on the drug market have not been met yet.

### 3.2. Resveratrol, Gut Microbiota, and Diabetes Mellitus

Alterations in microbiota composition can enhance heightened adiposity, which is linked to development of atherosclerosis, HTA, dyslipidemia, and type 2 diabetes mellitus (T2DM). Obesity is the most preventable, debilitating, and serious diet-related risk factor for the development of T2DM and CVD. It is still an important public health issue with an alarming incidence and tendency for further growth. However, results from many epidemiological studies suggest that dietary fibers significantly reduce the risk for obesity and associated chronic diseases, directly affecting the digestion, absorption, and appetite and indirectly through the microbiome [38]. Other additional mechanisms include a reduction in excess cholesterol and the inhibition of the bile acid reabsorption [39].

Moreover, modern lifestyles and nutrition along with genetic predisposition have increased the number of patients with T2DM in the last few decades. It is classified as an age-related disease and can affect the life span. Although it is not possible to avoid the development of T2DM in some cases, our habits may impact the onset of its appearance, for instance, the consumption of foods enriched in bioactive compounds and nutraceuticals, which means taking fruits, vegetables, and whole grains in increasing proportion during daily meals. It was observed that in some regions people were healthier and lived longer; thus, researchers’ attention was directed to the food they consumed and habits they had [40]. Among them, Mediterranean cuisine was particularly interesting, and consequential studies have indicated that these active nutrients had other biological properties, antioxidant, antiproliferative, antithrombogenic, anti-inflammatory, antiaging, antimicrobial, estrogenic, cardio-protective, and most importantly, antidiabetic. This effect was observed through increasing glucose metabolism and improving vascular function, as well as in reducing insulin resistance and the HbA1c level [41]. The glycemia-lowering effect of RSV was confirmed in both types of diabetes mellitus (type 1 and 2). This effect, also, may be the result of improved insulin sensitivity and the preservation of pancreatic beta cells [42]. In humans with insulin resistance, RSV improves glycemic control. By October 2023, 30 clinical trials were registered, available on www.clinicaltrials.gov (accessed on 25 September 2023) that relied on the effects of RSV on various forms of DM (type 1 and 2, gestational, and pre-diabetes, and its complications). Most of them were performed on T2DM patients; although, for many of them, the results could not be easily found. In the systematic review available in the Cochrane Database from 2020 [43], it has been suggested that randomized clinical trials that can support the efficacy of RSV supplementation in T2DM adult patients are still lacking. Possible mechanisms of RSV are correlated with SIRT1 expression in that RSV, through SIRT1, stimulates PPAR gamma coactivator 1-alpha (PGC1α) activity [44] and, through AMPK, regulates several important processes including energy metabolism and mitochondrial functions (it is known that AMPK activity is dysregulated in hyperglycemia) [45]. Additionally, RSV stimulates glucose uptake by increasing GLUT4 (glucose transporter type 4) expression in the plasma membrane of peripheral tissues. In a small open label study that included 13 patients with T1DM (youth and adults, both sexes) supplementation with 500 mg RSV twice daily over a period of two months led to significant and rapid reduction in the levels of fasting glycemia with a concomitant reduction in HbA1c and oxidative stress. The biochemical parameters were analyzed at three time points, at baseline and on the 30th and 60th days [46]. The new results published from a randomized placebo-controlled clinical trial performed on 472 elderly patients (older than 60 years) with T2DM, where the treatment group was administered 500 mg of RSV daily during the course of 6 months, showed that RSV significantly decreased the levels of HbA1c, C-reactive protein, and cytokines IL-6 (interleukin-6), TNF-α (tumor necrosis factor α), and IL-1β compared to the placebo [47]. T2DM is associated with several metabolic disorders including insulin resistance, hyperglycemia, and dyslipidemia, which play a main role in the development of diabetes complications. The long-term use of antidiabetic drugs (sulfonylureas and biguanides) is the main approach in the treatments of DM, but they cannot prevent progression of its complications [48]. The promising approach has been focused on natural compounds that can delay or prevent the onset of those complications, like RSV. Newer meta-analyses regarding the direct antidiabetic roles of RSV supplementation [49] showed a significant reduction in fasting blood glucose, insulin resistance, and glycated hemoglobin among diabetic patients, while its effects on reducing weight, body-mass index, waist circumference, and fat mass in obese patients, were also described [50]. 

**Figure 2:**
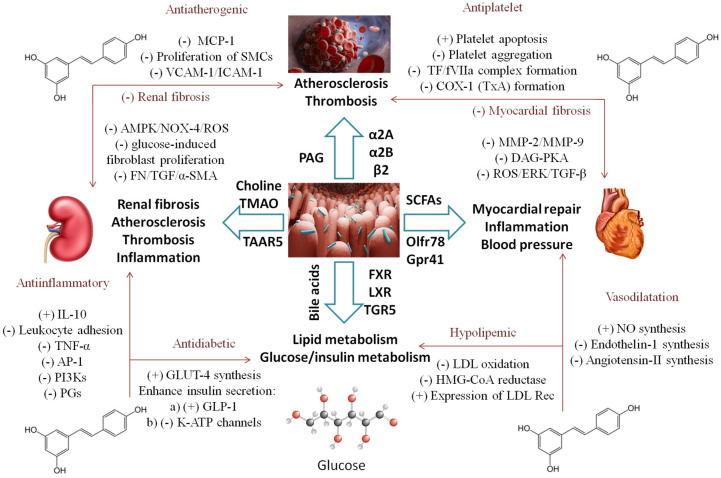
Synergistic action of the gut microbiome and RSV in the various processes of common CND (from Section 3.1 and Section 3.2). MCP-1—monocyte chemoattractant protein-1, SMC-smooth muscle cells, VCAM-1—vascular cell adhesion molecule-1, ICAM-1—intercellular cell adhesion molecule-1, TF—tissue factor, f VIIa—activated clotting factor VII, COX-1—cyclooxygenase-1, TxA—thromboxane A, AMPK—AMP-activated protein kinase, NOX-4—nicotinamide adenine dinucleotide phosphate oxidase 4, ROS—reactive oxygen species, α-SMA—α-smooth muscle actin, FN—cellular fibronectin, IL-10—interleukin 10, TNF—tumor necrosis factor, AP-1—activator protein 1, PI3Ks—phosphatidylinositol 3-kinases, PGs—prostaglandins, GLUT-4—glucose transporter 4, GLP-1—glucagon like peptide 1, K-ATP—ATP sensitive K channels, LDL—low density lipoprotein, HMG-CoA—3-Hydroxy-3-methylglutaryl-coenzyme A, NO—nitric oxide, MMP—matrix metalloproteinase, DAG-PKA—diacylglycerol-protein kinase A pathway, ERK—extracellular signal-regulated kinases, TGFβ—transforming growth factor beta, SCFAs—short-chain fatty acids, FXR/LXR—farnesoid/liver X receptors, TGR5—G protein coupled bile acid receptor, TAAR5—trace amine-associated receptor 5, Olfr-78—olfactory receptor-78, Gpr41—free fatty acid receptor, PAG—phenylacetylglutamine, TMAO—trimethylamine N-oxide, α/β—adrenergic receptors. (+) stimulates, (-) inhibits.

In a randomized, double-blind, and placebo-controlled trial of 56 patients with T2DM and coronary heart disease [51], a 4-week supplementation with 500 mg RSV daily turned out to be beneficial on glycemic control, HDL cholesterol, and the total-/HDL-cholesterol ratio, as well as in the total antioxidant capacity and a significant reduction in malondialdehyde levels [52]. In patients with T2DM, however, RSV failed to reduce waist circumference and triglyceride and HDL-cholesterol levels, according to the meta-analysis of 19 trials involving over 1100 patients [34]. The observed effects on the lipid profile are similar to those of the trials conducted on patients with metabolic syndrome, where, additionally, total serum cholesterol was significantly reduced. Also, there was no change in the liver enzyme activity, since the concentrations of alanine aminotransferase (ALT) [53] and aspartate aminotransferase (AST) [54] remained unchanged [55]. The lack of effect on liver enzyme levels, additionally including alkaline phosphatase (ALP), bilirubin, and gamma-glutamyl transferase (GGT) have also been confirmed in a pooled analysis of 15 randomized trials including over 700 participants [56]. On the other hand, in patients with nonalcoholic fatty liver disease (NAFLD), ALT and AST were significantly reduced [57], mostly in studies that lasted over 3 months and in patients with mean age < 45 years and BMI < 30kg/m^2^. RSV supplementation did not affect other metabolic indicators such as leptin and adiponectin levels [50]. Figure 2 represents phases in the pathogenesis of common CND where the RSV–gut-microbiota interaction could change the disease course and potential outcome.

### 3.3. Resveratrol, Gut Microbiota, and COVID-19 Infection in Patients with Preexisting Comorbidities

COVID-19 infection occurs in a wide clinical spectrum, ranging from asymptomatic patients to severe viral pneumonia, acute respiratory distress syndrome (ARDS), septic shock, and multiple organ failure [58]. Besides the ingested viral load, the presence of virulence factors, and previous prophylaxis, the favorable clinical outcome of the COVID-19 disease is associated mostly with the health status of a susceptible host; thus, a clinical course may vary among people with common comorbidities and previously healthy individuals. As a matter of fact, it seems that the hallmark of emerging diseases in the 2019–2022 period is represented by the interplay between viral particles and the metabolic and immunologic properties of a susceptible host. Finding a preferable treatment option for the complexity of this link sometimes requires the synergistic use of modern pharmacological treatment and traditional medicine–nutraceuticals, dietary fibers, and herbal supplements. Also, the current pandemic has shown the century in which we live imposes the need for more complex preventive and curative measures of chronic and stress-related diseases. After the observation that SARS-CoV-2 disrupts the gut–brain axis, which might lead to common disorders of gut–brain interaction, including functional dyspepsia and irritable bowel syndrome [59], experts have advised probiotic use to counteract the overall complications of COVID-19 infection [60]. 

During the pandemic, it was observed that increased mortality and worse outcomes took place among patients with comorbidities, especially with CVD and metabolic disorders caused by lifestyle (obesity, diabetes mellitus, etc.). Researchers’ logical option was RSV because it inhibited the release of proinflammatory cytokines, ameliorated vascular thrombosis, upregulated eNOS (endothelial NO-synthetase), enhanced endothelial NO production, reduced endothelin-1 synthesis, and inhibited oxidative stress—all of which could influence the complications.

RSV was included in clinical trials as supplementation for patients with a poor prognosis [61]. Additionally, RSV’s effects include inhibition of TLR4 (Toll-Like receptor 4) activation and inhibitions of proinflammatory transcription factor NF-κB and Th17 helper T-cells, which was promising in combating the COVID-19-mediated activation of TLR4 and the stimulation of proinflammatory cytokines (IL-1, IL-6, CCL-5 (chemotactic chemokine ligand 5)) and TNF-α [62,63]. A double-blind, randomized, and placebo-controlled proof-of-concept trial included 105 outpatients with mild COVID-19 infection [62]. All patients received supplementation with vitamin D3, while the treatment group received RSV as well. In the RSV treatment group, a lower incidence of hospitalization and pneumonia was observed. 

In patients with severe COVID-19 infections, the major cause of death was correlated with a cytokine storm and sepsis [64]. In an observational study of 230 patients with severe COVID-19 infections, supplementations with copper and RSV decreased the mortality rate twofold [65]. 

The main target of RSV distribution is the gastrointestinal tract; so, there is proof that RSV influences the gut microbiota, too. In eighteen studies (in vivo, ex vivo, in vitro) investigating the immune response, prevention of thromboembolic complications, and gene therapy after RSV supplementation, it was found that, upon administration, RSV changed the genetic expression in the microbial community. This mechanism could be suggested and applied in the treatment of disorders associated with microbial dysbiosis, like obesity, DM, degenerative diseases, and metabolic syndrome, as previously noted [13]. All of this can decrease the impact of morbidity (especially of CVD) in acute COVID-19 infection and in the second phase (post-COVID-19 syndrome) as well [61]. 

### 3.4. Resveratrol, Gut Microbiota, and Post-COVID-19 Syndrome

It is known that people with obesity/hypertension, metabolic syndrome, and/or immunodeficiency had worse outcomes regarding the consequences of the COVID-19 pandemic and a higher mortality rate [66,67]. This diverted investigation of the novel therapeutic strategies into dietary polyphenols and, shortly after, the gut microbiome. 

RSV was shown to modulate several steps in acute COVID-19 infection. The antiviral properties of RSV include the inhibition of viral replication through downregulation of several transcription factors and signaling pathways responsible for viral gene expression, nucleic acid, and protein synthesis. These activities have been proven for the treatment of several respiratory viruses, including SARS-CoV-2. The fact that low micromolar concentrations of RSV caused a 60–98% reduction in replication upon viral entry suggested that RSV interfered with the early phase of viral infection. When recovered from the acute disease, however, some patients might experience long-term symptoms that could last for months, in a so called ‘post-COVID-19 syndrome’. These include dyspnea, myalgia, fatigue, insomnia, cognitive disorders, and by far the most common, gastrointestinal disturbances (heartburn, abdominal pain, diarrhea, constipation) [68]. Recent findings suggest that gastrointestinal symptoms last 6 months after acute infection in 10–25% of patients and are commonly associated with some psychiatric disorders (anxiety and depression). The persistence of these symptoms is associated with a profound systemic inflammatory response as well as alteration of the gut microbiome [69].

In terms of preventing bacterial superinfection, the antimicrobial activity of RSV is also shown for both Gram-positive and Gram-negative bacteria and is mostly due to its ability to recruit and activate macrophages, neutrophils, and lymphocytes during the infection [70] and also to regulate gut microflora, enhancing the production of *Lactobacillus* sp. and *Bifidobacterium* spp. [70,71].

## 4. Resveratrol and Gut Microbiota in Healthy Individuals

### 4.1. Resveratrol, Gut Microbiota, and Physical Activity

Studies regarding RSV’s association with metabolic and cellular changes during physical activity, ergogenic properties, and an athlete’s performances are heterogeneous. RSV has been described as a calorie restriction mimetic; thus, its usage during physical activity may improve exercise performance [72]. Interestingly, an animal study with Wistar rats showed that the consumption of beverages rich in RSV during physical exercise improved health despite the consumption of a high-fat diet [73]. Other studies in mice [74,75,76] confirmed RSV’s effects on physical performance by significantly increasing the aerobic capacity. In a pilot randomized clinical trial of 60 elderly, it was indicated that the combination of RSV and exercise in this population is safe and may improve the mitochondrial function of skeletal muscle and mobility-related indices of physical function. Another double blind placebo-controlled clinical trial of 36 young untrained males indicated that supplementation with RSV before effective training had an impact on muscle pain reduction, making the recovery of the anaerobic capacity faster and reducing the frequency of muscle damage [77]. In both clinical trials, RSV was used at 500 and 1000 mg/day. However, in previous clinical trials [78,79], these exercise-induced positive effects in combination with RSV were not observed. A probable explanation was the low dose of RSV (250 mg/day). The pilot feasibility study performed in athletes who had run the London marathon also failed to confirm the benefits of RSV supplementation on the inflammatory response or delayed onset of muscle soreness [80], even though participants consumed 600 mg/day of RSV. However, the study included only seven healthy male athletes. A study of a course of 4 days of oral RSV supplementation in healthy athletes revealed no significant enhancement in the whole-body fat oxidation, muscle glycogen restoring, and mitochondria biosynthesis during the exercise period [81]. In correlation with this, participants involved in high-intensity cyclic training with the same duration and similar dose (480 mg/day) of RSV supplementation did not experience any changes in the exercise-induced fatigue, non-esterified fatty acid (NEFA), lactate dehydrogenases (LDH), uric acid (UA), and antioxidative (malondialdehyde, MDA) parameters. However, the concentration of IL-6 was significantly decreased; so, this could certainly benefit highly trained athletes [82].

The idea that RSV may have additional benefits when it is combined with physical activity lies in its capability to reduce oxidative stress [53]. Despite the fact that physical activity improves our health, it is well known that moderate exercise leads to oxidative stress, inflammation, and muscle injury. The hypothesis that RSV may diminish these side effects of exercise started to be the subject of research twenty years ago. Numerous animal studies have supported this hypothesis. In most of the studies, a dose of 10 mg/kg/day was found to be adequate to produce the benefits of RSV consumption during physical activity [53]. The beneficial effect of RSV during physical performance included prevention of muscle injury, a shorter time for recovery, improvement in muscle function, and an increase in exercise capabilities. All these positive effects of RSV on skeletal muscle are the results of the improvement in mitochondrial function and biogenesis by RSV [53]. In this regard, in healthy subjects with moderate to high intensity physical activity, oral RSV supplementation may improve maximum oxygen uptake (O_2max_) and diminish an abruptly released IL-6 response, which brings the novel ergogenic property of RSV into scientists’ research plan and empowers RSV benefits on healthy athletes’ performances.

### 4.2. Resveratrol, Gut Microbiota, and Quality of Life

Proper and continuous implementation of measures and activities within the domain of primary health care contributes to the reduction in numerous chronic and stress-related diseases but also improves quality of life (QoL) and overall psychosomatic public health. The measures of primary prevention include compliance with the principles of proper nutrition, good quality sleep, and regular moderate physical activity [83]. In addition, the presence of chronic diseases is not associated with increased mortality per se but with significant morbidity and alterations in daily activities. Those diseases include, for example, neurodegenerative diseases, chronic fatigue syndrome, fibromyalgia, inflammatory bowel disease, multiple sclerosis, rheumatoid arthritis, and, especially in younger people and adolescents, mild respiratory symptoms like allergic rhinitis and the common cold. RSV has the ability to enhance the effects of primary prevention measures, as well as to prevent or reduce the progression of diseases within different severity ranges, from mild to severe neurodegenerative diseases. Although there were discrepancies between animal studies and clinical trials [51] (since the initial studies showed no clear improvement in the cognitive function, mood, and sleep quality) in healthy, young humans [23], newer clinical trials succeeded in demonstrating that a single dose of 14 mg taken before sleep significantly improved non-REM sleep and the feeling of being well-rested compared to a placebo [84]. Some RSV-containing products even have been administered to patents in Japan for sleep improvement. A pilot study of people in the 65–93 age range with a sedentary lifestyle, including 90 days of RSV intake (1000 mg/day), showed a selective improvement in psychomotor speed [85] but no significant improvement in the cognitive function. On a larger scale, however, the improvement in the executive function and memory domain of elderly and postmenopausal women was observed in several studies, mostly when used daily for at least two weeks [86]. On a cellular level, this is explained by its involvement in multiple signaling pathways related to programmed cell death, cell survival, synaptic plasticity, as well as the activation of important processes such as sirtuin-mediated lifespan enhancement [87]. Along with the improvement in spatial working memory and hippocampal neurogenesis, RSV decreases the amyloid precursor protein; so, it can be safely used in the prevention of Alzheimer disease [51]. As for postmenopausal women, nutraceuticals containing RSV successfully enhanced postmenopausal symptoms (mood swings and cognitive performance), which were shown after 14 weeks of RSV supplementation (75 mg, twice daily). In a study of 60 perimenopausal women, RSV relieved neurovegetative symptoms (hot flushes, sleep disturbances, sweat, mood changes) improving their QoL and sexual life [88]. Another research group, on the other hand, reported no difference when consuming 75 mg of RSV twice a day for three months of postmenopausal women experiencing hormonal migraine, migraine-related disability, and migraine-associated QoL [27,89]. Since it has high affinity for the estrogenic receptor, there were concerns about the side effects on estrogen-sensitive tissues (e.g., breasts and endometrium). However, RSV intake has not been related to an increased risk for breast cancer and endometrial hyperplasia, according to clinical trials [90]. Moreover, a significant decrease in the frequency of angina episodes per week and the need for nitroglycerin were observed in the population after a 60-day treatment period [91], while RSV in combination with carboxymethyl-β-glucan as a nasal spray alleviated symptoms of allergic rhinitis and the common cold, such as rhinorrhea, nasal congestion, sneezing, sore throat, cough, and breathing difficulties [54]. The most important obstacle is the aforementioned low bioavailability of RSV after oral intake despite developing new carriers for RSV, including liposomal particles [92]. Further research also takes into consideration RSV precursors like polydatin and pterostilbene [93]. Despite numerous bioactive molecules that we ingest daily, the possible epigenetics effects of this have not been investigated yet. However, the effects of RSV on chromatin segregation and activation of the deacetylases of SIRTs makes this polyphenol more important than we thought. Through these processes, mitochondrial dysfunction, myocardial fibrosis, and vascular aging can be prevented [94]. Moreover, after translocation to the nucleus, SIRT1 may deacetylase transcription factor(s) directly or possibly epigenetically, deacetylating the corresponding histone proteins [95], with far reaching clinical benefits.

## 5. Conclusions

Based upon the available literature, the consumption of a diet enriched in plant polyphenols, especially RSV may redirect the natural course of noncommunicable diseases and act synergistically with other multimodal measures in enhancing overall health, including interference with human gut microbiota. Therefore, the daily use of a balanced Mediterranean diet is very useful, especially in predisposed people, with both modifiable and nonmodifiable risk factors. In that regard, this synergy can be helpful as well for the treatment of the long, deteriorating, and exhausting consequences of people recovering from COVID-19 infection, mostly due to multistep actions in complex post-COVID-19 syndrome. However, it is important to state that despite promising data, most of these are observational and insufficient. Further research on RSV-derived metabolites and gut microbiota changes is necessary in order to evaluate a deeper link between the products of this synergy, the immune system, and disease development, especially in immunocompromised people and patients with multiple comorbidities.
